# Patient and medication factors associated with preventable medication waste and possibilities for redispensing

**DOI:** 10.1007/s11096-018-0642-8

**Published:** 2018-05-02

**Authors:** C. L. Bekker, B. J. F. van den Bemt, A. C. G. Egberts, M. L. Bouvy, H. Gardarsdottir

**Affiliations:** 1 Department of Pharmacy, Sint Maartenskliniek, Hengstdal 3, 6574 NA Nijmegen, The Netherlands; 2 Department of Clinical Pharmacy, Division Laboratories and Pharmacy, University Medical Centre Utrecht, Heidelberglaan 100, 3584 CX Utrecht, The Netherlands; 3 Department of Pharmacy, Radboud University Medical Centre, Geert Grooteplein Zuid 10, 6525 GA Nijmegen, The Netherlands; 4 Department of Clinical Pharmacy and Toxicology, Maastricht University Medical Centre, P. Debyelaan 25, 6229 HX Maastricht, The Netherlands; 5 Division of Pharmacoepidemiology and Clinical Pharmacology, Utrecht Institute for Pharmaceutical Sciences, Utrecht University, 99, 3584 CG Utrecht, The Netherlands

**Keywords:** Community pharmacy, Medication waste, The Netherlands, Pharmacy services, Redispensing, Unused medications

## Abstract

*Background* Knowledge on factors related to preventable medication waste and waste-reducing interventions, including redispensing unused medications, is needed to maximise effectiveness. *Objective* To assess patient and medication factors associated with preventable medication waste and possibilities for redispensing unused medications. *Setting* Dutch community pharmacies. *Methods* In this cross-sectional study, pharmacy-staff registered patient and medication characteristics of prescription medications returned to 41 Dutch community pharmacies during 1 week in 2014. Medications were classified as preventable waste if the remaining amount could have been prevented and as theoretically eligible for redispensing if the package was unopened, undamaged and ≥ 6 months until the expiry date. Associations were analysed using multivariate logistic regression. *Main outcome measures* Proportion of medications classified as preventable waste and as eligible for redispensing, including factors associated with these medications. *Results* Overall, 279 persons returned 759 (low-cost) medications, and 39.3% was classified as preventable waste. These medications were more frequently used by men than women (OR; 1.7[1.2–2.3]) and by older (> 65 years) than younger patients (OR; 1.4[1.0–2.0]). Medications dispensed for longer periods were more often unnecessary wasted (1–3 months OR; 1.8[1.1–3.0], > 3 months 3.2[1.5–6.9]). Of all returned medications, 19.1% was eligible for redispensing. These medications were more frequently used by men than women (OR; 1.9[1.3–2.9]). Medications chronically used were more frequently eligible for redispensing than acute use (OR; 2.1[1.0–4.3]), and used for longer periods (1–3 months OR; 4.6[2.3–8.9], > 3 months 7.8[3.3–18.5]). *Conclusions* Over one-third of waste due to medications returned to community pharmacies can be prevented. One-fifth of returned medications can be redispensed, but this seems less interesting from an economic viewpoint.

**Electronic supplementary material:**

The online version of this article (10.1007/s11096-018-0642-8) contains supplementary material, which is available to authorized users.

## Impacts of Practice

- Numerous patients have leftover medication, of which almost half could have been prevented.
- Pharmacists can help to reduce medication waste by limiting medication quantities dispensed to patients and theoretically by redispensing medications that remain completely unused.
- The majority of medications that are returned to the pharmacies unused are of low value, making investments in redispensing unused medications less useful.

## Introduction

Medications account for almost one-fifth of health care spending in developed countries [1]. However, patients do not use a substantial proportion of medications dispensed to them [2–9**],** contributing to suboptimal treatment outcomes, financial waste and harm to the environment.

Various stakeholders in the medication supply chain, from manufacturer to patient, contribute to medication waste. Manufacturers may produce unnecessarily large packages with quantities that exceed the amount required for treatment. Pharmacists are not always allowed to split packages into smaller quantities and thus dispense excessive amounts to the patient. In addition, prescribers may prescribe medications for a longer period than the patient actually needs. Even if there is no waste of medication in the situations above, side effects, unsatisfactory treatment responses or early discontinuation during medication use may lead to therapy changes that may result in an excess of dispensed medication [3, 6–10]. Patients keep the remaining amounts for later use, discard them with the household garbage or return them to pharmacies and waste depots [11–15].

Although many studies have described which medications are returned to pharmacies and for which reasons, knowledge of the factors relating to medication waste is lacking [16]. If information were to be available on which medications are frequently unnecessary wasted, by which patients and in which situations, then waste-reducing interventions can specifically target these. Moreover, part of the medication waste often concerns unopened packages, including medications of good quality. A possible intervention to decrease the waste of these good quality medications might be the redispensing of these medications. In most countries, redispensing unused medications is not done in clinical practice due to lack of insight in the quality or legal restraints. However, the debate about redispensing as waste-reducing intervention is ongoing [17–21]. Therefore, more information is needed to assess which medications that remain unused by the patient could be eligible for redispensing.

## Aim of the study

The aim of this study is to assess which patient-related and medication-related factors are associated with preventable medication waste and to explore possibilities for redispensing unused medications.

## Ethics approval

The study was approved by the UPPER institutional review board of the Utrecht University, the Netherlands (UP1408).

## Method

### Design

This cross-sectional study was conducted in May 2014 in 41 Dutch community pharmacies that are part of the Utrecht Pharmacy Practice network for Education and Research (UPPER) of the department of Pharmaceutical Sciences of the Utrecht University [22]. The pharmacies were located in both urban and rural areas and covered 2.1% of the total number of community pharmacies (n = 1981).

### Data collection

Prescription medications that were returned as a routine practice to the participating community pharmacies during five consecutive working days in the study period were included in this study. Medications dispensed outside the Netherlands, extemporaneously compounded medications and medical devices such as wound dressings and testing materials were excluded. Pharmacy students holding a bachelor’s degree in pharmacy analysed the waste and collected the data during their final internship prior to receiving their pharmacy master’s degree. Students received both oral and written instructions before the start of the study. For each returned medication, a written record form was completed with information directly obtained from the person who returned the medication, after verbal consent and information derived from the medication label.

- The following patient characteristics were recorded anonymously: patient’s gender and age, type of prescriber (general practitioner, medical specialist, dentist or other), reason for use and reason(s) for returning the medication (patient deceased, condition resolved, passed expiration date, no/insufficient effect, treatment changed, adverse events, inconvenience of use, other [further specified] or unknown). Furthermore, details about the person who returned the medication (e.g. user, family, relative, health professional or other) were also registered.
- The following medication characteristics were recorded: medication name, strength, returned amount (number of tablets or capsules, liquids were estimated in milliliter, dermatologicals were estimated in grams), amount initially dispensed, prescribed dosage regimen, expiration date, whether the package was returned unopened (i.e. unused, yes/no) and whether the package was undamaged (yes/no). The returned medications were coded according to the Anatomical Therapeutic Chemical (ATC) classification system of the WHO [23].

Data were entered on site into the online survey tool Lime survey. The first author randomly checked 10% of the entered patients’ data sheets in terms of data entry and data validity. Data was considered as precise as less than 1% of all entered variables were found to be incorrect. Subsequently, the economic value of each individual returned medication was calculated by using the Dutch medication prices of May 2014 [24]. The lowest registered price of each medication unit was used to determine the minimal economic value of each returned medication unit. The total economic value was calculated by multiplying the returned number of units of a medication (e.g. number of tablets) by the unit price.

Each pharmacy received a unique study code. The study code list could only be accessed by an independent researcher who was not a member of the study group.

### Primary outcomes

All returned medications were classified according to their preventability of medication waste and eligibility for redispensing.

Firstly, predefined criteria were used to assess the preventability of the medication waste. This assessment was done by the pharmacy student who collected the data. This assessment was based on the patient- and medication information and subsequently judged on preventability when one of the following criteria was full filled: (I) when larger amounts of medication were prescribed than needed for the expected duration of use, (II) when excessive medication amounts were prescribed for a terminal patient, (III), when a pharmacist dispensed more than the prescribed amount, (IV) in case of a prescription error (e.g. wrong strength prescribed), (V) when a refill that was no longer needed was dispensed or (VI) when patients had side effects or insufficient effect of treatment at the moment of a refill, but still collected the medication. Medications that could be classified neither as preventable waste nor as inevitable waste, due to insufficient data that was not registered, were excluded from further analysis.

Secondly, the returned medications were classified theoretically as eligible for redispensing when these met all of the following criteria: (I) the package was unopened, (II) the package was undamaged, and (III) there was at least 6 months between the date of returning (end of study date) and the expiry date.

### Analysis

Regarding proportions, descriptive analyses were made and expressed as percentages, whereas medians with interquartile ranges (IQR) were analysed for averages. A univariate analysis was initially conducted in order to assess potential associations between explanatory variables and the primary outcomes waste (yes/no) and eligibility for redispensing (yes/no). This was followed by a full model multivariate logistic regression analysis. Explanatory variables included in both analyses were patient’s gender and age, reason for returning the medication, duration of use (determined by a clinical pharmacologist), unit price and amount dispensed (converted into days by dividing by the daily dose). In addition, regarding medication waste, the prescriber of the returned medication was also included but not considered as a potential association for medications’ eligibility for redispensing.

The definition of waste could have been biased by the subjective judgement of the student who collected the medications at the pharmacy. Therefore, to enhance validity a sensitivity analysis was conducted to which a returned medication classified as waste at a certain pharmacy was matched to a returned medication classified as no waste at the same pharmacy. Conditional logistic regression, with controlling for the pharmacy level, was subsequently applied. All statistical analyses were performed in STATA13.

## Results

### Characteristics of the returned medications and users

In total, 279 persons returned 759 prescription medications. Medications were most often returned by the consumer (59.9%), followed by a family member (31.5%). The returned medications were most frequently used for gastro-intestinal disorders (18.5%), nervous system disorders (17.8%) and cardiovascular disorders (18.1%).

The estimated total economic value of all returned medications was €7,916 with a median value of €1.75 per medication (IQR €0.58–6.28). Of the ten most expensive returned medications, half were considered eligible for redispensing (Appendix, Table 1).

Medications were returned primarily because ‘patient was deceased’ (22.4%), ‘condition had resolved’ (19.9%) and ‘passed expiry date’ (14.6%) Some patients reported ‘other’ reasons such as discontinuation of treatment during pregnancy, switching to a multi-dose dispensing system or ‘spring-cleaning’ of the house. The main reasons for returning medications that were eligible for redispensing were ‘patient was deceased’ (30.3%) and ‘treatment changed’ (19.3%) (Table 1).

**Table 1:** Patients’ reasons for returning the medication

Reasons for returning	All medication n = 759* (%)	Medication eligible for redispensing n = 145* (%)
Patient was deceased	170 (22.4)	44 (30.3)
Condition had resolved	151 (19.9)	17 (11.7)
Passed expiry date	111 (14.6)	–
Other	81 (10.7)	23 (15.9)
No/insufficient effect	73 (9.6)	18 (12.4)
Treatment changed	68 (9.0)	28 (19.3)
Unknown	54 (7.1)	5 (3.5)
Adverse events	55 (7.3)	12 (8.3)
Inconvenience of use	10 (1.3)	1 (0.7)

*More than one answer possible

### Factors associated with preventable medication waste

Of the 759 returned medications, 298 medications (39.3%) were classified as preventable medication waste and 378 medications (49.8%) were classified as inevitable waste. Due to a lack of information, 83 medications could not be classified and were therefore excluded from the analysis. Medications classified as preventable waste were distributed among all therapeutic classes, and had an average economic value of €2.36 (IQR €0.72–9.00). Around 80% of the preventable medication waste was below €15.00. Factors that were associated with potential preventable medication waste are presented in Table 2.

**Table 2:** Factors associated with preventable medication waste

Medication waste	Preventable n = 298 (%)	Inevitable n = 378 (%)	Crude OR (95% CI)	Adjusted OR (95% CI)
Patient related				
Gender				
Female	155 (52.0)	243 (64.3)	Ref	Ref
Male	139 (46.6)	129 (34.1)	**1.7 (1.2**–**2.3)**	**1.7 (1.2**–**2.3)**
Unknown	4 (1.3)	6 (1.6)	–	–
Age				
0–65	135 (45.3)	212 (56.1)	Ref	Ref
> 65	159 (53.4)	160 (42.3)	**1.7 (1.2**–**2.4)**	**1.4 (1.0**–**2.0)**
Unknown	4 (1.3)	6 (1.6)	–	–
Medication related				
Prescriber				
General practitioner	163 (54.7)	191 (50.5)	Ref	Ref
Medical specialist	107 (35.9)	127 (33.6)	1.2 (0.8–1.8)	0.9 (0.6–1.3)
Unknown	28 (9.4)	60 (15.9)	–	–
Reasons for returning				
Condition resolved	56 (18.8)	89 (23.5)	Ref	Ref
Adverse events	26 (8.7)	27 (7.1)	1.5 (0.8–2.9)	1.3 (0.7–2.5)
No/insufficient effect	32 (10.7)	35 (9.3)	1.5 (0.8–2.6)	1.3 (0.7–2.3)
Patient was deceased	48 (16.1)	97 (25.7)	0.8 (0.5–1.3)	0.6 (0.4–1.1)
Other	118 (39.6)	126 (33.3)	1.5 (1.0–2.3)	1.5 (1.0–2.4)
Unknown	18 (6.0)	4 (1.1)	–	–
Duration of use				
Acute	47 (15.8)	77 (20.4)	Ref	Ref
Chronic	157 (52.7)	171 (45.2)	1.5 (1.0–2.3)	1.1 (0.7–1.8)
Episodic	94 (31.5)	130 (34.4)	1.2 (0.8–1.9)	1.1 (0.7–1.9)
Price unit				
€0–1	257 (86.2)	306 (81.0)	Ref	Ref
€1–5	22 (7.4)	32 (8.5)	0.8 (0.5–1.4)	0.7 (0.4–1.3)
> €5	15 (5.0)	36 (9.5)	**0.5 (0.3**–**0.9)**	0.6 (0.3–1.1)
Unknown	4 (1.3)	4 (1.2)	–	–
Amount dispensed				
0–14 days	64 (21.5)	102 (27.0)	Ref	Ref
15–30 days	70 (23.5)	113 (29.9)	1.0 (0.6–1.5)	1.0 (0.6–1.6)
1–3 months	95 (31.9)	87 (23.0)	**1.7 (1.1**–**2.7)**	**1.8 (1.1**–**3.0)**
> 3 months	25 (8.4)	15 (4.0)	**2.7 (1.3**–**5.4)**	**3.2 (1.5**–**6.9)**
Unknown	44 (14.8)	61 (16.1)	–	–

Significant associations are shown in bold

Preventable waste was significantly higher among male patients compared to female patients (OR 1.7 [1.2–2.3]). Medications used by older patients (> 65 years) were classified as preventable waste significantly more often than medications that were originally in use by younger patients (< 65 years) (OR 1.4 [1.0–2.0]). The type of prescriber, type of medication use, reason for returning the medication and the economic value of a medication unit were not significantly associated with medications defined as preventable waste. However, a significantly increased risk of preventable medication waste was found for medications that were initially dispensed for a longer period (1-3 months OR 1.8 [1.1–3.0] and > 3 months OR 3.2 [1.5–6.9]). Sub analyses showed that approximately one-third of the medications used on a chronic basis and two-thirds of the episodic medications were dispensed for less than 1 month.

The conditional logistic regression showed similar associations, except for two variables that turned out to be significant: reason for returning ‘other’ (OR 1.9 [1.1–3.4]) and medication units valued €1–5 (OR 0.3 [0.1–0.7]) (Appendix, Table 2).

### Factors associated with medications eligible for redispensing

Of all of the returned medications, 145 medications (19.1%) were classified theoretically as eligible for redispensing, with a median economic value of €4.60 (IQR €1.45–17.36). Around 80% of the returned medications were below €25.00. Factors that were associated with medications potentially eligible for redispensing are presented in Table 3.

**Table 3:** Factors associated with medication eligible for redispensing

Redispensing	Eligible n = 145 (%)	Not eligible n = 614 (%)	Crude OR (95% CI)	Adjusted OR (95% CI)
Patient related				
Gender				
Female	67 (46.2)	388 (63.2)	Ref	Ref
Male	78 (53.8)	214 (34.9)	**2.1 (1.5**–**3.0)**	**1.9 (1.3**–**2.9)**
Unknown	0 (–)	12 (2)	–	–
Age				
0–65	56 (38.6)	328 (53.4)	Ref	Ref
> 65	89 (61.4)	274 (44.6)	**2.3 (1.4**–**3.7)**	1.3 (0.9–2.0)
Unknown	0 (–)	12 (2.0)	–	–
Medication related				
Reasons for returning				
Condition resolved	17 (11.7)	132 (21.5)	Ref	Ref
Adverse events	12 (8.3)	42 (6.8)	2.2 (1.0–5.0)	1.7 (0.7–4.1)
No/insufficient effect	15 (10.3)	53 (8.6)	**2.2 (1.0**–**4.7)**	1.5 (0.7–3.5)
Patient was deceased	44 (30.3)	125 (20.4)	**2.7 (1.5**–**5.0)**	1.6 (0.8–3.2)
Other	54 (37.2)	242 (39.4)	1.7 (1.0–3.1)	1.3 (0.7–2.4)
Unknown	3 (2.1)	20 (3.3)	–	–
Duration of use				
Acute	12 (8.3)	128 (20.9)	Ref	Ref
Chronic	102 (70.3)	273 (44.5)	**4.0 (2.1**–**7.5)**	**2.1 (1.0**–**4.3)**
Episodic	31 (21.4)	213 (34.7)	1.6 (0.8–3.1)	1.6 (0.8–3.4)
Price unit				
€0–1	121 (83.5)	514 (83.7)	Ref	Ref
€1–5	13 (9.0)	45 (7.3)	1.2 (0.6–2.3)	1.6 (0.8–3.4)
> €5	11 (7.6)	47 (7.7)	1.0 (0.5–2.0)	1.5 (0.7–3.3)
Unknown	0 (–)	8 (1.3)	–	–
Amount dispensed				
0–14 days	14 (9.7)	163 (26.6)	Ref	Ref
15–30 days	22 (15.2)	172 (28.0)	1.5 (0.7–3.0)	1.3 (0.6–2.6)
1–3 months	78 (53.8)	130 (21.2)	**7.0 (3.8**–**12.9)**	**4.6 (2.3**–**8.9)**
> 3 months	20 (13.8)	23 (3.8)	**10.1 (4.5**–**22.8)**	**7.8 (3.3**–**18.5)**
Unknown	11 (7.6)	126 (20.5)	–	–

Significant associations are shown in bold

Medications classified as eligible for redispensing were used by male patients significantly more frequently compared to female patients (OR 1.9 [1.3–2.9]). Medications used on a chronic basis were more frequently eligible for redispensing compared to acute use (OR 2.1 [1.0–4.3]). Of the returned medications that were initially dispensed for a longer period, significantly more medications were eligible for redispensing (1–3 months OR 4.6 [2.3–8.9] and > 3 months OR 7.8 [3.3–18.5]). The other variables showed no association with medications eligible for redispensing.

## Discussion

This study showed that of the returned medications, more than one-third was perceived as preventable waste. This emphasizes the need to implement waste reducing measures where possible. Moreover, approximately one-fifth of the returned medications were potentially eligible for redispensing. This study also identified several patient- and medication-related factors that were associated with preventable waste and possibilities for redispensing.

Male gender was associated with preventable medication waste. Previous research showed that men more frequently use medications intended for chronic use (like cardiovascular diseases), whereas women more often use medications that are used for acute or episodic treatment (like antibiotics, painkillers and sleeping pills) [25, 26]. When assessing the association between the dispensed amount and preventable waste, medications dispensed for a duration exceeding one month were associated with preventable waste. This has also been confirmed by others [27] and indicates that preventable waste depends strongly on the amount of medications dispensed. Furthermore, returned medications classified as waste were more often used by the elderly. An explanation could be that the elderly often use multiple medications, which increases the risk of non-adherence, side-effects and eventually waste [28].

The proportion of medications that was theoretically eligible for redispensing is similar to that reported by others [9, 29]. One study found that more than 90% of returned medications were eligible for redispensing, but this study did not apply the criterion that the original outer package must be unopened and intact [30]. However, none of those studies examined determinants of returned medications that are eligible for redispensing. This study shows that eligibility of returned medications for redispensing was specifically associated with male users, chronic therapy duration and a dispensing period of at least one month. To obtain the most benefit from redispensing if implemented in clinical practice, interventions can be specifically designed for medications that are dispensed to male users, and medications that are used on a chronic basis or dispensed for at least one month. Medications dispensed for longer periods more often consist of multiple packages. Therefore it is more likely that at least one package is left unopened and thus eligible for redispensing. This also indicates that interventions for redispensing unused medications should include patients to whom multiple packages of a medication are dispensed. To make redispensing feasible to implement in practice, multiple stakeholders have reported that patients should be willing to participate in such a system [17]. Redispensing unused medications may succeed if patients are willing to return all their unused medications to the pharmacy, and even more important, are willing to use medications that have been previously dispensed to another patient. In an internet hotline launched by the Dutch Ministry of Health where patients and health care professionals were asked to report on how to combat waste in healthcare, the majority of suggestions made by patients were to redispense unused medications [31]. Hence, this suggests that patients are willing to participate in a redispensing system.

Knowing this, waste reducing interventions should specifically target the amount that is dispensed to patients, such as dispensing medications for shorter periods, which has proven to be effective in reducing waste [32]. However, implementing this approach for all medications might not compensate for the reimbursement of additional dispensing fees by pharmacists. In specific cases of more expensive medications, it may be cost-effective to shorten the dispensing period. Our results showed that the most expensive returned medications consisted of large amounts (Appendix, Table 1). Similarly, it is questionable if the redispensing of unused medications is cost saving for all medications. Nevertheless, there are also benefits to be gained by reducing environmental harm. Reducing medication waste at community pharmacies, where the majority of patients use relatively cheap generic medications, requires a multifactorial and medication-specific approach [33]. For example, thoroughly reviewing the medication for older patients, and discussing which medications are needed, could decrease the risk of medications being wasted.

To assess the effectiveness of waste-reducing interventions, studies are needed that assess if changing dispensing from a 3-month to a 1-month supply reduces waste and saves costs, taking into account the low costs of the returned medications. In addition, patients’ views on a supply of one month should be determined, as this requires more pharmacy visits and may be a burden to patients. Little research is conducted on redispensing unused medications. Insight into the costs of a redispensing system is needed to determine if implementation is cost-beneficial in the community and/or outpatient pharmacy. Furthermore, patients’ views on the redispensing of unused medications should be explored in terms of their willingness to use medications that have been dispensed to another patient.

### Limitations

In this study, students subjectively determined if medications were defined as preventable waste, which may limit validity. To enhance validity of this data, student received both oral and written instructions about this classification, with a clear set of criteria. Regarding all data that the students collected, and the personal communication that they had with the persons returning the medications, they were, in our view, best able to make this judgement. This judgement was not reviewed by a second person. In our view, a review of the classifications later on and using the data sheets only would have been less precise compared to the assessment made on site. Furthermore, a sensitivity analysis was conducted that corrected for each pharmacy, i.e. the student that made the judgement in the pharmacy in the analysis. For instance, it may have been that a student more frequently classified returned medications as preventable waste. This analysis presented similar findings on factors that were associated with preventable medication waste, indicating that there was no ‘inter-pharmacy’ variety in classifications.

Three criteria were used to determine if the medications were potentially eligible for redispensing (package unopened, intact and at least six months until the expiry date). However, no information about the home storage conditions, like temperature exposure, was taken into account. Literature has shown that patients do not always store their medications at the recommended temperature [34]. This might affect the quality of medications and thereby patient safety. Therefore, the proportion of medications that was considered of good quality and eligible for redispensing in this study is likely an overestimation. Further, we found that redispensing unused medications that are returned to community pharmacies is less feasible when considering the small proportion deemed eligible and the low costs of these medications.

No collection campaign was set up prior to the start of this study. Knowing that not all patients return their medications to the pharmacy, but that they also deposit these at chemical waste depots, keep them in the house or dispose of them with the garbage, the absolute extent of waste generated through community pharmacies could not be assessed.

Furthermore, using the lowest medication price unit for the calculations might have resulted in an underestimation of the economic value. For many returned medications, information was lacking on the number of packages that were returned. Medications classified as eligible for redispensing could consist of unopened and opened packages, which might have caused an overestimation of the economic value of these medications. Finally, in the Netherlands, the majority of expensive medications, such as most biologicals, are dispensed by hospital based outpatient pharmacies. These medications are infrequently returned to community pharmacies.

## Conclusion

This study shows that over one-third of the waste due to medications returned to the community pharmacies can be prevented. Waste-preventive interventions could specifically target factors that are associated with preventable medication waste, such as the dispensing of medications for period longer than one month. Approximately one-fifth of returned medications can be redispensed. However, most medications were of low-cost, which makes redispensing unused medications in the community pharmacy less interesting from an economic point of view.

## Electronic supplementary material

Below is the link to the electronic supplementary material.

Supplementary material 1 (DOCX 22 kb)
