# Supplementary material for: Patient and medication factors associated with preventable medication waste and possibilities for redispensing
Source: Int J Clin Pharm. 2018 May 2;40(3):704–11. doi: 10.1007/s11096-018-0642-8 (PMC5984955; doi:10.1007/s11096-018-0642-8)
Supplement: Supplementary file 1 — Supplementary material 1 (DOCX 22 kb) [file 11096_2018_642_MOESM1_ESM.docx]

**Appendices**

Table 1: The ten most costly returned medications, the reasons for returning and if they were classified as medication waste and/or eligible for redispensing

| Medication (number of returned units) | Economic value (€) | Reason for returning | Preventable waste | Eligible for redispensing* |
| --- | --- | --- | --- | --- |
| Fentanyl 50 mcg spray 40 doses (3) | 726.00 | Patient was deceased |  |  |
| Ondansetron 16 mg suppository (34) | 476.00 | Patient was deceased |  |  |
| Follitropin alpha 900IE/1.5 ml injection (1) | 354.00 | Unknown |  |  |
| Ketanserin 20 mg tablet (168) | 196.56 | Condition resolved |  |  |
| Methylphenidate 54 mg tablet with controlled release (90) | 180.00 | Switch from brand to generic, but switched back to brand variant by prescriber |  |  |
| Insulin detemir 100 IE/ml injection (15) | 165.00 | Therapy changed |  |  |
| Pregabalin75 mg capsule (128) | 128.34 | Patient was deceased |  |  |
| Eplerenone 25 mg tablet (61) | 124.44 | No/insufficient effect |  |  |
| Oxycodone 40 mg tablet with controlled release (60) | 117.60 | Patient was deceased |  |  |
| Tiotropium bromide 18 mcg inhalation capsules (68) | 98.60 | Therapy changed |  |  |

**Medications were partly eligible for redispensing, as not all returned packages were unopened*

Table 2: Conditional logistic regression, with controlling for the pharmacy level, on factors associated with preventable medication waste. Significant associations are shown in bold

| Medication waste | Preventable n=245 (%) | Inevitable n=245 (%) | Crude OR  (95% CI) | Adjusted OR (95% CI) |
| --- | --- | --- | --- | --- |
| Patient related |  |  |  |  |
| Gender  Female  Male  Unknown | 120 (49.0)  121 (49.4)  4 (1.6) | 151 (61.6)  88 (35.9)  6 (2.5) | Ref  **1.9 (1.3-2.9)**  - | Ref  **1.8 (1.2-2.9)**  - |
| Age  0-65  >65  Unknown | 103 (42.0)  138 (56.3)  4 (1.6) | 132 (53.9)  107 (43.7)  6 (2.5) | Ref  **1.8 (1.2-2.7)**  - | Ref  **1.6 (1.1-2.5)**  - |
| Medication related |  |  |  |  |
| Prescriber  General practitioner  Medical specialist  Unknown | 122 (49.8)  98 (40.0)  25 (10.2) | 129 (52.7)  85 (34.7)  31 (12.7) | Ref  1.3 (0.8-1.9)  - | Ref  1.4 (0.8-2.2)  - |
| Reasons for returning  Condition resolved  Adverse events  No/insufficient effect  Patient was deceased  Other  Unknown | 45 (18.4)  18 (7.4)  28 (11.4)  41 (16.7)  103 (42.0)  10 (4.1) | 57 (23.3)  19 (7.8)  26 (10.6)  62 (25.3)  78 (31.8)  3 (1.2) | Ref  1.3 (0.6-2.8)  1.5 (0.7-2.9)  0.8 (0.4-1.4)  **1.8 (1.1-3.1)**  - | Ref  1.0 (0.4-2.2)  1.2 (0.5-2.5)  0.6 (0.3-1.1)  **1.9 (1.1-3.4)**  - |
| Duration of use  Acute  Chronic  Episodic | 39 (15.9)  135 (55.1)  71 (29.0) | 48 (19.6)  100 (40.8)  97 (39.6) | Ref  **1.7 (1.1-2.9)**  0.9 (0.5-1.5) | Ref  1.0 (0.6-1.8)  0.8 (0.5-1.4) |
| Price unit  €0-1  €1-5  >€5  Unknown | 217 (88.6)  13 (5.3)  13 (5.3)  2 (0.8) | 193 (78.8)  25 (10.2)  25 (10.2)  2 (0.8) | Ref  **0.5 (0.2-0.9)**  **0.5 (0.2-0.9)**  - | Ref  **0.3 (0.1-0.7)**  0.5 (0.2-1.0)  - |
| Amount dispensed  0-14 days  15-30 days  1-3 months  >3 months  Unknown | 52 (21.2)  56 (22.9)  80 (32.7)  21 (8.6)  36 (14.7) | 64 (26.1)  79 (32.2)  45 (18.4)  9 (3.7)  48 (19.6) | Ref  0.9 (0.5-1.5)  **2.2 (1.3-3.8)**  **2.7 (1.2-6.5)**  - | Ref  1.0 (0.6-1.8)  **2.4 (1.3-4.3)**  **3.0 (1.2-7.6)**  - |
